# Development and Validation of a Machine Learning–Based Model of Mortality Risk in First-Episode Psychosis

**DOI:** 10.1001/jamanetworkopen.2024.0640

**Published:** 2024-03-18

**Authors:** Johannes Lieslehto, Jari Tiihonen, Markku Lähteenvuo, Stefan Leucht, Christoph U. Correll, Ellenor Mittendorfer-Rutz, Antti Tanskanen, Heidi Taipale

**Affiliations:** 1 Department of Forensic Psychiatry, Niuvanniemi Hospital, University of Eastern Finland, Kuopio; 2 Division of Insurance Medicine, Department of Clinical Neuroscience, Karolinska Institutet, Stockholm, Sweden; 3 Center for Psychiatry Research, Stockholm City Council, Stockholm, Sweden; 4 Department of Clinical Neuroscience, Karolinska Institutet, Stockholm, Sweden; 5 Department of Psychiatry and Psychotherapy, School of Medicine, Technical University of Munich, Munich, Germany; 6 The Zucker Hillside Hospital, Department of Psychiatry, Northwell Health, Glen Oaks, New York; 7 Department of Psychiatry and Molecular Medicine, Donald and Barbara Zucker School of Medicine at Hofstra/Northwell, Hempstead, New York; 8 Department of Child and Adolescent Psychiatry, Charité–Universitätsmedizin Berlin, Berlin, Germany; 9 School of Pharmacy, University of Eastern Finland, Kuopio

## Abstract

**Importance:**

There is an absence of mortality risk assessment tools in first-episode psychosis (FEP) that could enable personalized interventions.

**Objective:**

To examine the feasibility of machine learning (ML) in discerning mortality risk in FEP and to assess whether such risk predictions can inform pharmacotherapy choices.

**Design, Setting, and Participants:**

In this prognostic study, Swedish nationwide cohort data (from July 1, 2006, to December 31, 2021) were harnessed for model development and validation. Finnish cohort data (from January 1, 1998, to December 31, 2017) were used for external validation. Data analyses were completed between December 2022 and December 2023.

**Main Outcomes and Measures:**

Fifty-one nationwide register variables, encompassing demographics and clinical and work-related histories, were subjected to ML to predict future mortality risk. The ML model’s performance was evaluated by calculating the area under the receiver operating characteristic curve (AUROC). The comparative effectiveness of pharmacotherapies in patients was assessed and was stratified by the ML model to those with predicted high mortality risk (vs low risk), using the between-individual hazard ratio (HR). The 5 most important variables were then identified and a model was retrained using these variables in the discovery sample.

**Results:**

This study included 24 052 Swedish participants (20 000 in the discovery sample and 4052 in the validation sample) and 1490 Finnish participants (in the validation sample). Swedish participants had a mean (SD) age of 29.1 (8.1) years, 62.1% were men, and 418 died with 2 years. Finnish participants had a mean (SD) age of 29.7 (8.0) years, 61.7% were men, and 31 died within 2 years. The discovery sample achieved an AUROC of 0.71 (95% CI, 0.68-0.74) for 2-year mortality prediction. Using the 5 most important variables (ie, the top 10% [substance use comorbidities, first hospitalization duration due to FEP, male sex, prior somatic hospitalizations, and age]), the final model resulted in an AUROC of 0.70 (95% CI, 0.63-0.76) in the Swedish sample and 0.67 (95% CI, 0.56-0.78) in the Finnish sample. Individuals with predicted high mortality risk had an elevated 15-year risk in the Swedish sample (HR, 3.77 [95% CI, 2.92-4.88]) and an elevated 20-year risk in the Finnish sample (HR, 3.72 [95% CI, 2.67-5.18]). For those with predicted high mortality risk, long-acting injectable antipsychotics (HR, 0.45 [95% CI, 0.23-0.88]) and mood stabilizers (HR, 0.64 [95% CI, 0.46-0.90]) were associated with decreased mortality risk. Conversely, for those predicted to survive, only oral aripiprazole (HR, 0.38 [95% CI, 0.20-0.69]) and risperidone (HR, 0.38 [95% CI, 0.18-0.82]) were associated with decreased mortality risk.

**Conclusions and Relevance:**

In this prognostic study, an ML-based model was developed and validated to predict mortality risk in FEP. These findings may help to develop personalized interventions to mitigate mortality risk in FEP.

## Key Points

**Question:**

Is it feasible to develop a machine learning model that can predict mortality risk in first-episode psychosis?

**Findings:**

This prognostic study developed and validated a machine-learning model using extensive Swedish and Finnish databases, identifying mortality risk in first-episode psychosis. For patients with predicted high risk, only long-acting injectable antipsychotics and mood stabilizers were associated with decreased mortality risk; among those with predicted low risk, oral aripiprazole and risperidone were associated with decreased mortality risk.

**Meaning:**

If further validated, this model may help to develop personalized interventions to mitigate mortality risk in first-episode psychosis.

## Teaser

This prognostic study evaluates the use of machine learning to predict mortality risk in first-episode psychosis and assesses whether these predictions can inform pharmacotherapy choices.

## Introduction

Mortality risk for individuals experiencing their first episode of psychosis (FEP) is several-fold higher than that of the general population.^1,2,3^ Moreover, epidemiologic research underscores the disproportionately high susceptibility of mortality due to suicidal behavior, injuries, and poisoning.^2^ Individuals with schizophrenia are more likely to succumb to somatic deaths (eg, due to cardiovascular incidents or malignant neoplasms) over the duration of the disorder compared with the general population.^1,4^

Previous epidemiologic research has outlined factors associated with elevated mortality risk in psychotic disorders, such as substance abuse, nonadherence to antipsychotics, and psychotic symptoms.^1,5,6^ Despite a solid epidemiologic understanding, we lack predictive models to identify premature mortality. Building such a model requires comprehensive, unselected, longitudinal data collection endeavors, enabling a detailed examination of individual risk trajectories across a long follow-up time. A pertinent question is what variables should constitute a model for mortality risk in FEP, given the complexity and heterogeneity of the disorder and its course.^7,8^ Machine learning (ML) algorithms have emerged as a promising solution for such data mining tasks due to their ability to predict individual patient outcomes by analyzing many predictor variables and their intricate, high-dimensional interactions.^9^ To our knowledge, no previous studies have used ML to predict mortality risk in FEP.

A prognostic tool could help to develop personalized treatment, reducing the mortality gap between FEP and the general population. Although oral antipsychotics remain the predominant first-line pharmacotherapy for FEP in clinical practice,^10^ long-acting injectable (LAI) antipsychotics are associated with a 33% mortality risk reduction when juxtaposed with their oral counterparts in schizophrenia.^11^ One lingering question is whether LAI antipsychotics should be broadly prescribed to all patients with FEP instead of oral agents or whether their use could be optimized by targeting specific patient risk profiles. Current clinical guidelines recommend LAI antipsychotics for instances of nonadherence or when favored by the patient, but they provide no direction regarding further stratification.^12,13^

Here, we developed an ML model for mortality risk in FEP using large, unselected nationwide register databases. Our overarching aim was to build an ML model based on a small set of variables that could be assessed in routine clinical work within a few minutes, akin to the risk calculators used in other fields of medicine.^14,15,16^ We also examined whether the ML model–based risk groups have differences in associations between the use of specific pharmacotherapies and mortality risk over 15 years after FEP. For these purposes, we developed and validated an ML model using a nationwide Swedish cohort of patients with FEP and externally validated its generalizability using an independent Finnish cohort.

## Methods

### Study Design and Data Acquisition

For this prognostic study, we used 2 nationwide register datasets from Sweden and Finland with identical exclusion criteria (eMethods in Supplement 1). Ethical permission for this research project was granted by the Regional Ethics Board of Stockholm. The Finnish National Institute for Health and Welfare, the Social Insurance Institution of Finland, and Statistics Finland also granted permission. Because this study was registry based without direct contact with participants, informed consent was not required by Swedish and Finnish legislation. Throughout our study, we followed the Transparent Reporting of a Multivariable Prediction Model for Individual Prognosis or Diagnosis (TRIPOD) reporting guideline.

The Swedish cohort consisted of individuals with first-episode nonaffective psychosis diagnoses (*International Statistical Classification of Diseases, Tenth Revision* [*ICD-10*], codes F20-F29) who were aged 15 to 45 years and registered treatment contact in Sweden between July 1, 2006, and December 31, 2021. The Swedish cohort was identified from the National Patient Register (inpatient and specialized outpatient care) and the MiDAS Register (disability pensions and sick leave). The Finnish cohort comprised individuals aged 16 to 45 years who received inpatient care for first-episode schizophrenia (available data for *ICD-10* codes F20 and F25) in Finland from 1998 to 2014, and were followed between January 1, 1998, and December 31, 2017. The Finnish cohort was identified from the Hospital Discharge Register maintained by the Finnish Institute for Health and Welfare. Both samples had a 1-year washout period for antipsychotics (ie, no antipsychotic treatment) before diagnosis to ensure the composition of true FEP cases.

### Statistical Analysis

#### ML Analysis

We trained an ML model to predict mortality risk immediately after a FEP diagnosis. We used a large variety of clinical, sociodemographic, and socioeconomic variables based on all register sources available during 1 or 2 years before FEP diagnosis in the Swedish cohort (51 variables, detailed in eTable 1 in Supplement 1) to predict 2-year mortality (vs survival) after FEP diagnosis. Initially, we used all 51 variables, with a subsequent refinement focusing on the top 10% (5 variables) to enhance applicability. A 2-year follow-up served as our inclusion criterion, optimizing the dataset size (eFigure 1 in Supplement 1), although some participants had data for up to 15 years. Despite its calibration for 2-year mortality, the overarching objective was a comprehensive mortality risk model; consequently, we evaluated the model’s predictive capability across the entire available follow-up (≤15 years in the Swedish sample and ≤20 years in the Finnish sample). The variables were retrieved from the National Patient Register (clinical history), the MiDAS Register (work-related history, disability pensions, and sick leave), the National Prescribed Drug Register (previous pharmaceutical treatments), and the Swedish Longitudinal Integrated Database for Health Insurance and Labour Market Studies (demographic variables). To train the ML model, we first randomly split the Swedish sample into a discovery sample and a validation sample. The model was trained using eXtreme Gradient Boosting (XGBoost; XGBoost Contributors^17^) in a nested cross-validation framework in the discovery sample (details in eMethods in Supplement 1) using R, version 4.1.1 (R Project for Statistical Computing). We selected XGBoost over other ML algorithms due to its superior performance in analyzing tabular data.^18^

We then identified the 5 most important variables (ie, top 10%) and retrained a model using these variables in the discovery sample. This model was then applied to the Swedish and Finnish validation samples. The contributions of the variables to the model’s predictions are presented as Shapley Additive Explanation (SHAP) values, which are described in more detail elsewhere.^19^ Briefly, SHAP is a game theoretic approach that helps delineate the directional association (positive or negative) of each variable within the ML model, enhancing insight into the model’s decision-making process. We also assessed model calibration using the Brier score and the calibration slope (ie, alignment between predictions and true outcome probabilities). The recalibration model was trained in the discovery sample and applied to the Swedish and Finnish validation samples. We also created Kaplan-Meier survival curves for the predicted groups over the total follow-up.

#### Pharmacoepidemiologic Analysis

In the discovery sample, we investigated whether the associations of different pharmacotherapies (with antipsychotics as the main exposure) and mortality risk varied between patients predicted to die after FEP diagnosis and those predicted to survive up to 15 years of follow-up after FEP. As supplementary analyses, we also conducted these pharmacoepidemiologic analyses without the ML-based stratification. For these purposes, we conducted between-individual Cox regression analyses using SAS, version 9.4 (SAS Institute Inc), for all available follow-up (≤15 years). The models were adjusted for time, concomitant pharmacotherapy and medications used to treat substance use disorders (SUDs; Anatomical Therapeutic Chemical [ATC] codes N07BB and N07BC), and sequence of antipsychotics. We investigated the following pharmacotherapies: antipsychotics (ATC code N05A excluding lithium N05AN01), antidepressants (ATC code N06A), mood stabilizers (ATC codes N03AF01, N03AG01, N03AX09, and N05AN01), and benzodiazepines and similar compounds (ATC codes N05BA, N05CD, and N05CF). We obtained the drug usage periods for time-varying exposure by analyzing prescription drug purchases using the prescription drug purchases to drug use periods (PRE2DUP) method, which has been described elsewhere.^20^ The PRE2DUP method calculates sliding averages of defined daily doses, drug purchase amounts, and individual drug use patterns and also takes into account hospital stays and medicine stockpiling of drugs.

Statistical significance was set at *P* < .05 (2-tailed). Data analyses were completed between December 2022 and December 2023.

## Results

### Group-Level Sociodemographic and Clinical Differences at Baseline

We gathered data on 24 052 patients with FEP from the Swedish cohort (20 000 in the discovery sample and 4052 in the validation sample) and 1490 patients (in the validation sample) with FEP from the Finnish cohort, as presented in the Table. The Swedish cohort had a mean (SD) age of 29.1 (8.1) years, and more than half (62.1%) were men. A total of 418 Swedish patients died within 2 years (69 due to natural causes and 281 to unnatural causes). There were 27 individuals receiving disability pensions or on sick leave at baseline. The mean (SD) follow-up of Swedish patients was 8.4 (3.9) years. The Finnish cohort had a mean (SD) age of 29.7 (8.0) years, and more than half (61.7%) were men. A total of 31 Finnish patients died within 2 years (3 due to natural causes and 28 to unnatural causes). The mean (SD) follow-up of Finnish patients was 14.2 (4.5) years. The Finnish cohort comprised only those treated with inpatient care, whereas 70.0% of the Swedish cohort were diagnosed in outpatient care. Compared with the Swedish sample, the Finnish cohort included only patients with either schizophrenia or schizoaffective disorder.

**Table.** Clinical and Sociodemographic Characteristics of the Study^a^

Characteristic	Swedish discovery sample (n = 20 000)				Swedish validation sample (n = 4052)				Finnish validation sample (n = 1490)			
	Died within 2 y (n = 350)	Survived (n = 19 650)	*t*/χ^2^/*z*	*P* value	Died within 2 y (n = 68)	Survived (n = 3984)	*t*/χ^2^/*z*	*P* value	Died within 2 y (n = 31)	Survived (n = 1459)	*t*/χ^2^/*z*	*P* value
Age, mean (SD), y	31.2 (7.92)	29.0 (8.10)	*t* = 5.19	<.001	30.7 (7.40)	29.0 (8.10)	*t* = 1.8	.07	29.50 (8.61)	29.70 (7.97)	*t* = −0.08	.93
Sex												
Male	269 (76.9)	12 168 (61.9)	χ^2^ = 31.98	<.001	51 (75.0)	2440 (61.2)	χ^2^ = 4.78	.03	26 (83.9)	893 (61.2)	χ^2^ = 5.67	.02
Female	81 (23.1)	7482 (38.1)			17 (25.0)	1544 (38.8)			5 (16.1)	566 (38.8)		
Education, y^b^												
Elementary, ≤9	126 (36.0)	6037 (30.7)	χ^2^ = 9.83	.007	23 (33.8)	1236 (31.0)	χ^2^ = 3.51	.17	NA	NA	NA	NA
High school, 10-12	157 (44.9)	7578 (38.6)			34 (50.0)	1456 (36.5)			NA	NA		
University or college, >12	51 (14.6)	3949 (20.1)			10 (14.7)	836 (21.0)			NA	NA		
Any employment (in year before FEP)	129 (36.9)	6690 (34.1)	χ^2^ = 1.09	.30	20 (29.4)	1346 (33.8)	χ^2^ = 0.39	.53	NA	NA	NA	NA
Receiving disability pension at baseline	60 (17.1)	2417 (12.3)	χ^2^ = 6.99	.008	9 (13.2)	498 (12.5)	χ^2^ = 0	>.99	NA	NA	NA	NA
On sick leave at baseline	19 (5.5)	1504 (7.7)	χ^2^ = 2.11	.15	<5	301 (7.6)	*z* = 0.23	.76	NA	NA	NA	NA
Duration of first hospitalization, median (range),	5 (1-95)	2 (0-100)	*z* = 5.03	<.001	3 (0-87)	2 (0-100)	*z* = 1.04	.30	38 (1-95)	45 (0-100)	*z* = −1.67	.09
Place of FEP diagnosis												
Inpatient care	163 (46.6)	7138 (36.3)	χ^2^ = 15.14	<.001	31 (45.6)	1454 (36.5)	χ^2^ = 2.01	.16	31 (100)	1459 (100)	χ^2^ = 0	>.99
Outpatient care	187 (53.4)	12 512 (63.7)			37 (54.4)	2530 (63.5)			0	0		
FEP diagnosis, *ICD-10* code												
Schizophrenia, F20	18 (5.1)	1144 (5.8)	χ^2^ = 6.78	.24	6 (8.8)	234 (5.9)	*z* = 0.68	.49	25 (80.7)	1200 (82.3)	χ^2^ < 0.001	>.99
Delusional disorder, F22	30 (8.6)	1968 (10.0)			<5	410 (10.3)			0	0		
Brief psychotic disorder, F23	150 (42.9)	7224 (36.8)			24 (35.3)	1423 (35.7)						
Schizoaffective disorder, F25	5 (1.4)	440 (2.2)			0	92 (2.3)			6 (19.4)	259 (17.8)		
Unspecified psychosis, F29	138 (39.4)	8179 (41.6)			33 (48.5)	1687 (42.3)			0	0		
Other psychotic disorder	9 (2.6)	695 (3.5)			<5	138 (3.5)			0	0		
Substance use comorbidities (within year before FEP)												
Alcohol related	35 (10.0)	819 (4.2)	χ^2^ = 27.20	<.001	7 (10.3)	155 (3.9)	χ^2^ = 5.57	.02	<5	73 (5.0)	*z* = 0.43	.67
Opioid related	27 (7.7)	331 (1.7)	χ^2^ = 67.73	<.001	5 (7.4)	56 (1.4)	χ^2^ = 12.19	<.001	0	18 (1.2)	*z* < 0.001	>.99
Cannabis related	14 (4.0)	700 (3.6)	χ^2^ = 0.09	.77	<5	125 (3.1)	*z* < 0.001	>.99	0	15 (1.0)	*z* < 0.001	>.99
Stimulant related	23 (6.6)	359 (1.8)	χ^2^ = 38.82	<.001	<5	66 (1.7)	*z* = 1.61	.11	<5	21 (1.4)	*z* = 1.75	.08
Multiple drug use	78 (22.3)	1367 (7.0)	χ^2^ = 118.27	<.001	17 (25.0)	238 (6.0)	χ^2^ = 37.88	<.001	<5	29 (2.0)	*z* = 1.50	.13

Abbreviations: FEP, first-episode psychosis; *ICD-10*, *International Statistical Classification of Diseases, Tenth Revision*; NA, not available.

^a^ Unless indicated otherwise, values are presented as No. (%) of patients.

^b^ Information missing for some individuals.

### ML Results

**Figure 1.**
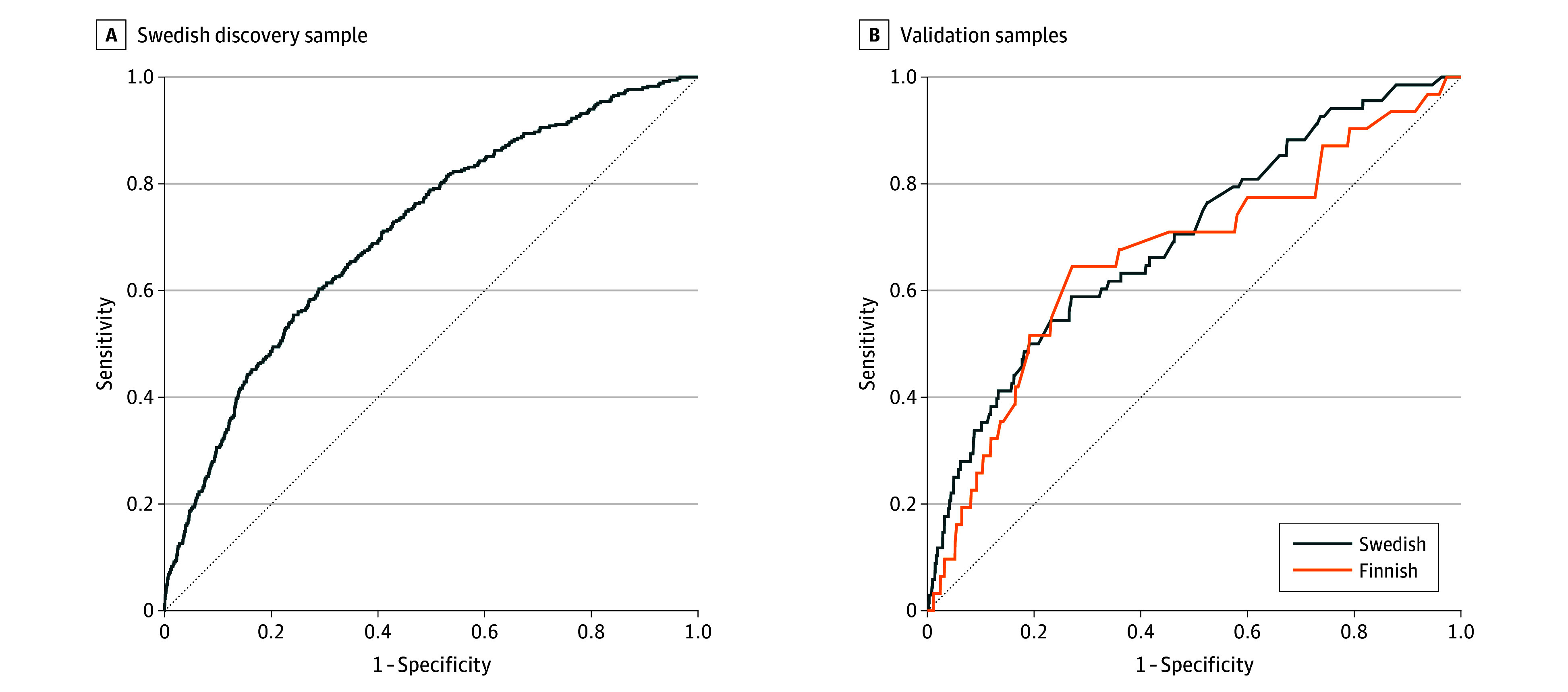
Receiver Operating Characteristic (ROC) Curves for the Prediction of 2-Year Mortality in First-Episode Psychosis (FEP) A, Out-of-training classification of 2-year mortality vs survival in the Swedish discovery sample using 51 variables. B, Prediction of 2-year mortality in the Swedish and Finnish validation samples using the final model based on 5 variables (previous substance use disorder comorbidities, duration of first hospitalization due to FEP, male sex, number of previous somatic hospitalizations, and age) trained in the discovery sample. Dashed lines indicate random classification.

The out-of-training classification of mortality within 2 years in the Swedish model discovery sample (n = 20 000) resulted in an area under the receiver operating characteristic curve (AUROC) of 0.71 (95% CI, 0.68-0.74; *P* < .001), with 59.7% sensitivity and 71.3% specificity (Figure 1A). In this analysis, we included post-FEP variables such as pharmacotherapy within 30 days and early outpatient visits that were used as a proxy for treatment plans at the time of FEP diagnosis because we did not have access to patient records. However, a reanalysis excluding these variables yielded similar performance, with an AUROC of 0.70, 58.3% sensitivity, and 71.4% specificity. Of the different causes of death (eFigure 2 in Supplement 1), the best performance was observed for substance-related deaths, including accidental substance- or drug-related poisonings (80.7% accuracy) and any substance- or drug-related death (73.8% accuracy). Among suicides, the best performance was observed for substance- or drug-related suicides (63.8% accuracy) and the worst for suicide by hanging (42.9% accuracy). Among the 5858 patients predicted to die, 2-year mortality was 5.0% without antipsychotic treatment (n = 1153) vs 3.2% with treatment (n = 4705) (χ^2^ = 8.40, *P* = .004). Among the 19 650 individuals with 2-year predicted survival, those hospitalized for a suicide attempt within 2 years had a higher likelihood of being falsely predicted by the model to die (vs predicted survival) (odds ratio [OR], 2.12 [95% CI, 1.86-2.42]; *P* < .001). The same was true for patients with a hospitalization for somatic reasons compared with those without any hospitalizations for somatic reasons over the 2-year follow-up (OR, 1.93 [95% CI, 1.80-2.07]; *P* < .001). The predictive performance for mortality over the 15 years after FEP among the 590 patients with available follow-up data aligned with the aforementioned 2-year mortality predictions (AUROC, 0.69 [95% CI, 0.61-0.78]).

**Figure 2.**
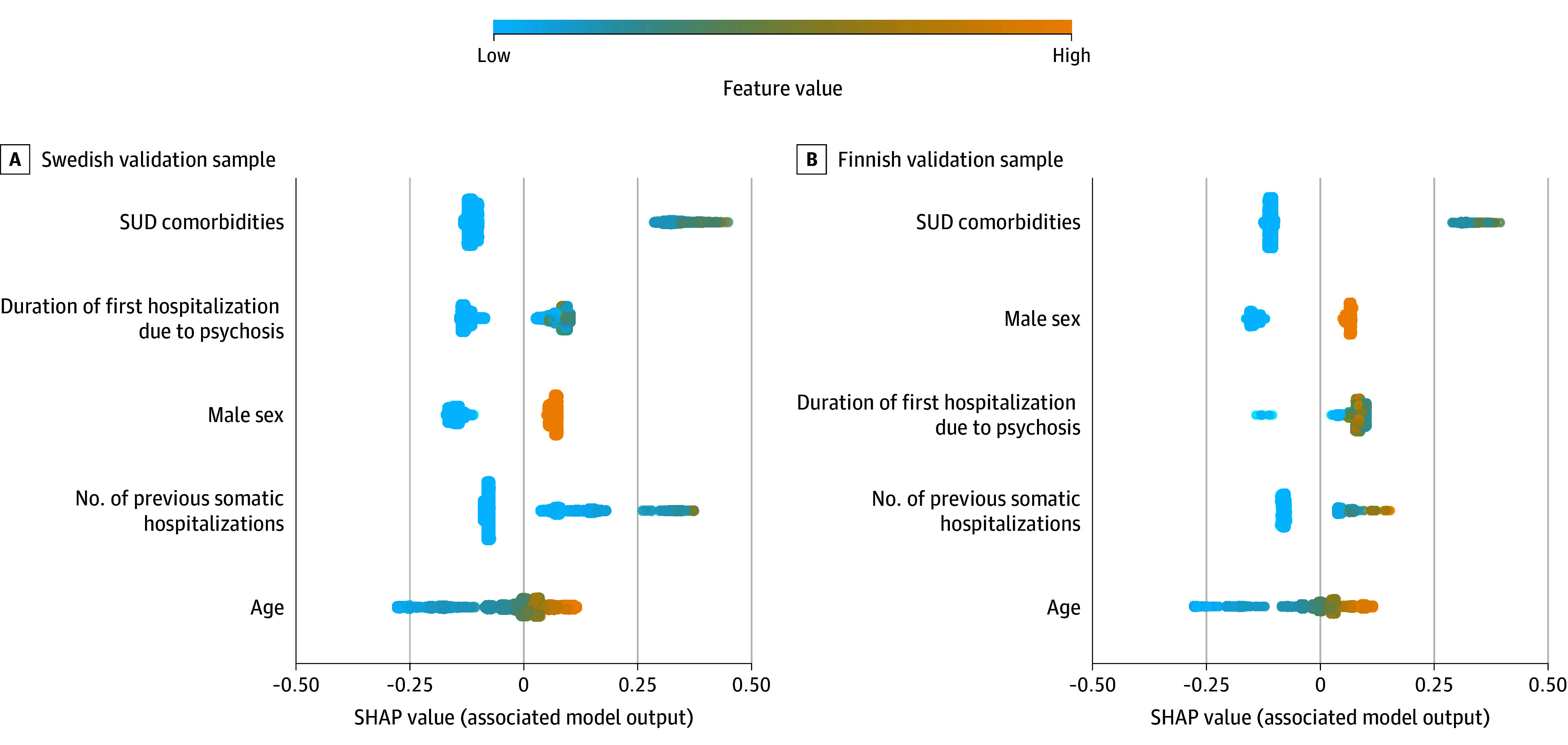
Final Model Shapley Additive Explanation (SHAP) Value Contributions Swedish (A) and Finnish (B) validation samples. For each of the 5 variables (features), the SHAP values for each patient with first-episode psychosis (FEP) are displayed horizontally, with color coding indicating the magnitude of these values. The variables are ranked by their mean absolute SHAP value (ranked from the most important to the least from top to bottom). Each point on the chart represents a patient with FEP for a specific variable. The x-axis indicates whether the variable was positively or negatively associated with the model’s prediction of mortality. The color scheme illustrates the variable’s value, adjusted according to the range observed in the dataset (ie, high values in warm colors and low values in cool colors). For example, being male is assigned a high value by this variable, which is positively associated with the model’s prediction of higher mortality risk. SUD indicates substance use disorder.

The variables most important to the model are shown in eTable 1 in Supplement 1. We retrained an ML model in the discovery sample using only the topmost important variables (substance use comorbidities, first hospitalization due to FEP, male sex, prior somatic hospitalizations, and age) and tested its performance for 2-year mortality prediction (Figure 1B) in the Swedish validation sample (AUROC, 0.70 [95% CI, 0.63-0.76]; *P* < .001; 54.4% sensitivity and 76.2% specificity) and the Finnish validation sample (AUROC, 0.67 [95% CI, 0.56-0.78]; *P* = .002; 64.5% sensitivity and 72.0% specificity). A prediction of death was associated with a high number of previous SUD comorbidities, long duration of first hospitalization due to FEP, male sex, a high number of previous somatic hospitalizations, and older age in both samples (Figure 2). The model showed good calibration in the Swedish validation sample (Brier score, 0.02; slope, 0.97) and the Finnish validation sample (Brier score, 0.02; slope, 0.97; eFigure 3 in Supplement 1). An online version of the model, Mortality Risk Assessment Calculator for Early First Episode Psychosis (MIRACLE-FEP), is available.^21^

**Figure 3.**
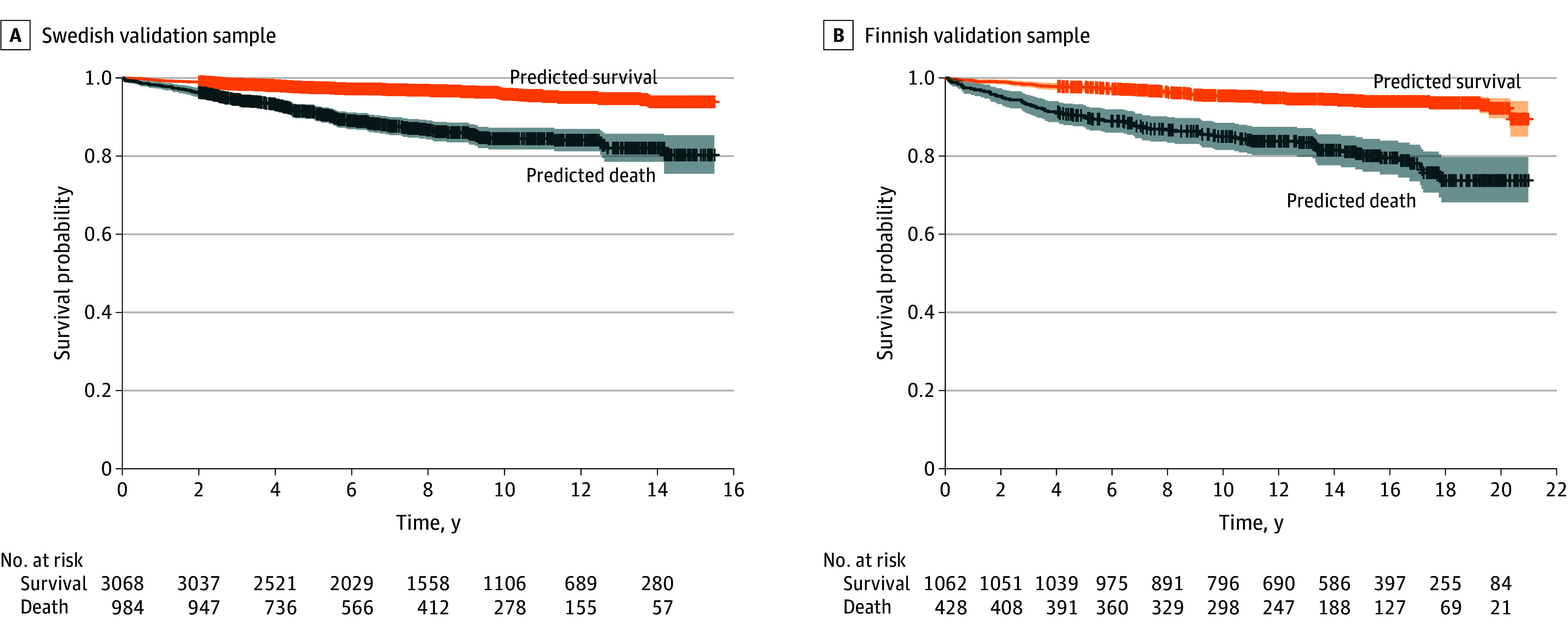
Survival Analyses Kaplan-Meier plots are shown for Swedish (A) and Finnish (B) validation samples with total follow-up (time in years) and censoring. Stratification was done using the final model with 5 variables trained in the discovery sample: substance use comorbidities, first hospitalization duration due to first-episode psychosis, male sex, prior somatic hospitalizations, and age. Shaded areas represent 95% CIs.

Mortality predictive performance of the model over 15 years in the Swedish validation sample and over 20 years in the Finnish validation sample aligned with the aforementioned 2-year mortality predictions (eTable 2 in Supplement 1). Kaplan-Meier curves for model predictions for the total available follow-up in the Swedish and Finnish validation samples are presented in Figure 3. In the Swedish validation sample, the 15-year survival rate among patients predicted to die was 80.3% (95% CI, 75.5%-85.3%) vs 93.9% (95% CI, 92.4%-95.3%) for those predicted to survive (HR, 3.77 [95% CI, 2.92-4.88]; *P* < .001). In the Finnish validation sample, the 20-year survival rate was 73.7% (95% CI, 68.2%-79.7%) for patients predicted to die vs 92.2% (95% CI, 89.7%-94.8%) for those predicted to survive (HR, 3.72 [95% CI, 2.67-5.18]; *P* < .001).

### Pharmacoepidemiologic Results

**Figure 4.**
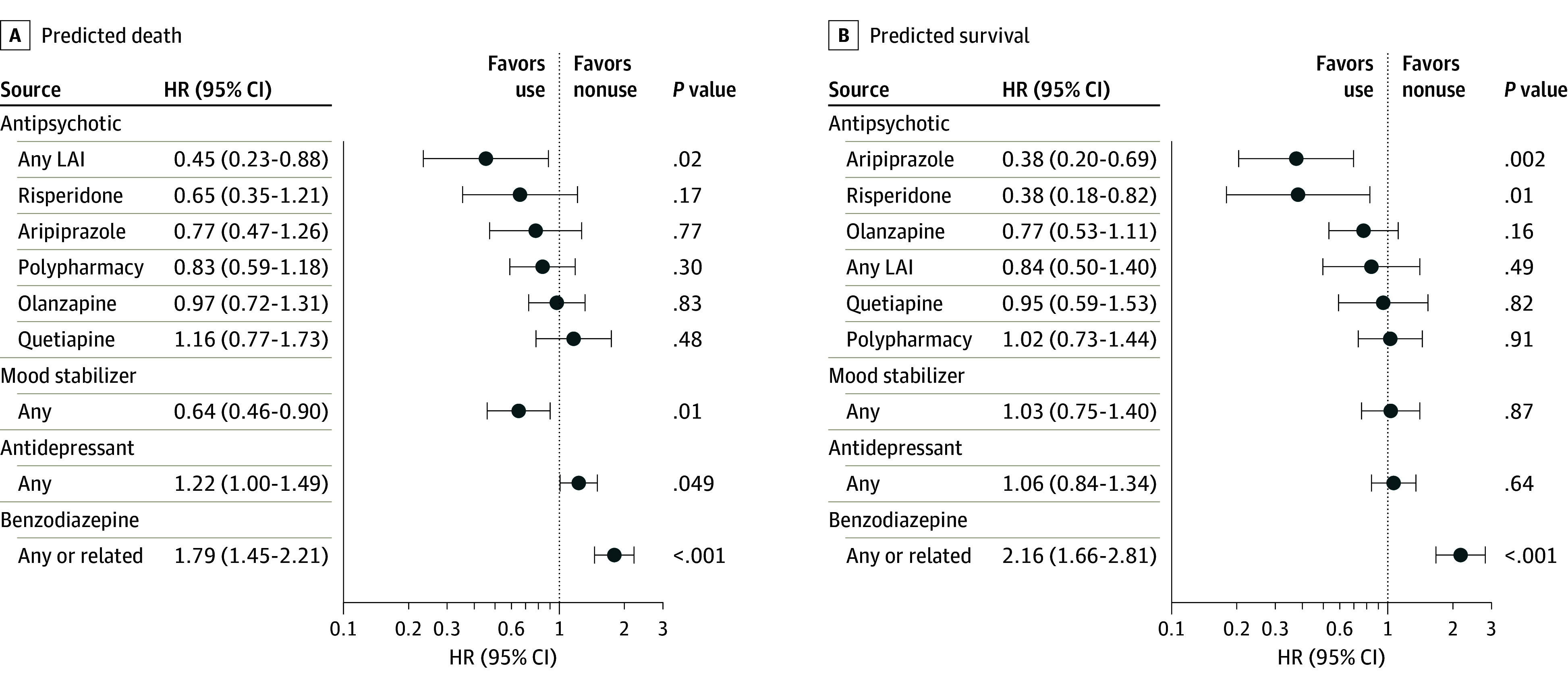
Associations of Different Pharmacotherapies and Risk of Death Over the 15-Year Follow-Up A, Patients predicted to die (5858 [29.3%] in the whole sample). B, Patients predicted to survive (14 142 [70.7%]). Analyses were stratified based on the out-of-training predictions in the discovery sample (n = 20 000). Antipsychotics were oral except for any long-acting injectable (LAI), which includes all LAI antipsychotics. Polypharmacy refers to concomitant use of 2 or more antipsychotics. HR indicates hazard ratio.

We used ML predictions to stratify patients by their predicted outcome. In examining 15-year mortality risk associated with different pharmacotherapies, we found that the rank order of the most effective treatments varied between groups (Figure 4). The use of LAI antipsychotics (HR, 0.45 [95% CI, 0.23-0.88]; *P* = .02 compared with nonuse) and mood stabilizers (HR, 0.64 [95% CI, 0.46-0.90]; *P* = .01 compared with nonuse) was associated with decreased mortality risk among patients who were predicted to die (ie, high-risk) within 2 years (5858 [29.3%]), but not among those predicted to survive (14 142 [70.7%]). The use of LAI antipsychotics over the follow-up did not differ between those predicted to die and those predicted to survive (χ^2^ = 0.11, *P* = .74) (eTable 3 in Supplement 1). Among those predicted to survive (ie, low risk), oral aripiprazole (HR, 0.38 [95% CI, 0.20-0.69]; *P* = .01) and oral risperidone (HR, 0.38 [95% CI, 0.18-0.82]; *P* = .002) were the only agents associated with decreased mortality risk over time compared with nonuse of antipsychotics. Benzodiazepine use was associated with increased mortality risk in both patients predicted to die (HR, 1.79 [95% CI, 1.45-2.21]; *P* < .001) and those predicted to survive (HR, 2.16 [95% CI, 1.66-2.81]; *P* < .001) (eTable 4 in Supplement 1). Without ML-based stratification in the whole discovery sample (eFigure 4 in Supplement 1), oral risperidone (HR, 0.46 [95% CI, 0.29-0.75]; *P* = .002), oral aripiprazole (HR, 0.49 [95% CI, 0.33-0.71]; *P* = .002), and LAI antipsychotics (HR, 0.64 [95% CI, 0.43-0.96]; *P* = .03) were the only pharmacologic treatments associated with statistically significantly lower mortality risk.

## Discussion

Using extensive Swedish and Finnish nationwide databases, we thoroughly developed and validated^22^ an ML model to predict future mortality risk among patients with FEP. The final model, which is available online,^21^ was based on just 5 variables: previous SUD comorbidities, duration of first hospitalization due to FEP, male sex, number of previous somatic hospitalizations, and age. This parsimony, without substantial performance sacrifice, and scalability to assess these variables quickly and cost-effectively is crucial for clinical applicability. The model’s discrimination performance (AUROC, 0.70) parallels risk calculators used in other medical disciplines (eg, surgery and cardiology^14,15,16^), providing robust generalizability and good calibration. After 2 decades following FEP diagnosis, the model’s predictions equated to about 30.0% of deaths in the group with high mortality risk, in contrast with less than 8.0% among those predicted to survive.

Our model effectively flagged patients with a high risk of poor outcomes, particularly those with SUD comorbidities, confirming the known negative effects of SUD on recovery from psychosis.^5,23^ The model reached its utmost predictive accuracy for substance- and drug-related deaths, accurately identifying two-thirds of substance-induced suicides. Consequently, effective interventions addressing SUD comorbidities are crucial in potentially mitigating the associated mortality risks in this high-risk group. Note, however, that the model failed to identify suicides not induced by substances or drugs. Although the model produced several false-positive predictions for the 2-year follow-up, a closer analysis indicated that these individuals exhibited more hospitalizations for somatic conditions and suicide attempts within this time frame. This underlines the need for a holistic approach in treating individuals at risk, addressing potential health complications and self-harm tendencies even if immediate mortality risk appears overstated, at least for the subsequent 2 years after FEP diagnosis.

Although the model is promising, augmentation with additional modalities could enhance its performance. There is a substantial inclination toward using high-cost modalities like neuroimaging or genetics in psychiatric ML research.^24,25,26^ However, relying on these more expensive and less accessible modalities is not financially prudent for widespread risk assessments in FEP, even if they may offer enhanced prognostic precision. A pragmatic approach would be to commence screening with a model utilizing readily accessible variables, allocating high-cost methodologies for uncertain cases requiring more refined prognostic accuracy. Previous research indicates the feasibility of achieving cost-efficient workflows by integrating clinician risk estimates with predictions from multimodal ML algorithms.^25^ Nevertheless, pursuing a flawless model remains elusive in clinical practice, with clinicians already addressing a portion of the mortality risk (eg, through pharmacotherapeutic and psychosocial inventions), thereby affecting the model’s perceived predictive performance. In fact, among individuals predicted to die, we observed a substantially higher 2-year mortality rate among those not receiving antipsychotic medication compared with their counterparts taking medication.

In disciplines like oncology, treatment is routinely stratified to individual clinical profiles.^27^ In this context, we examined the association between the use of pharmacotherapies and mortality in different ML model–based risk groups. We found that among individuals with predicted high mortality risk, the use of LAI antipsychotics and mood stabilizers after FEP diagnosis was associated with decreased mortality risk over the subsequent 15 years. However, these associations were not observed among patients predicted to survive. For the latter group, only the use of oral aripiprazole and oral risperidone was associated with decreased mortality risk during the same period. The effectiveness of LAI antipsychotics, recognized as pivotal in enhancing medication adherence,^28^ underscores the importance of ensuring their availability to individuals at heightened mortality risk. The effectiveness of mood stabilizers might stem from their ability to mitigate impulsivity, a known risk factor for impulsive suicidal behavior and accidents.^29^ Finally, in both prediction groups, benzodiazepine use was associated with increased mortality risk, which suggests a need for careful consideration when using these medications in FEP.

Conventional FEP treatment protocols may delay optimal treatment response in approximately 23% of individuals with treatment-resistant symptoms present at illness onset,^30,31^ due to a lack of refined tools for identifying this subgroup. Without ML-based stratification (eFigure 4 in Supplement 1), a broad recommendation of oral aripiprazole or risperidone might seem logical given the association of these medications with the lowest mortality risk. Nevertheless, our findings suggest that approximately 30.0% of patients with FEP might need an alternative therapeutic approach, because a proportion of this subgroup may have treatment-resistant symptoms from the onset. Early initiation of LAIs and mood stabilizers is likely critical for this high-risk subgroup, whereas oral antipsychotics might be adequate as first-line treatment for the majority of patients. However, it is crucial to acknowledge that some patients with low mortality risk may still require treatment with LAI antipsychotics when adherence is poor.

### Strengths and Limitations

This study has both strengths and limitations. A primary strength is the harnessing of extensive, unselected nationwide databases encompassing 20 000 patients with FEP for model creation and subsequent validation in a distinct 1490-patient cohort from another country with a slightly different health care system and follow-up period. Consequently, our model could be generalizable to cohorts within similar demographics within government-funded health care systems. However, our dataset lacked granularity in specific clinical metrics, including psychotic symptomatology and pharmacotherapeutic indications, which might have augmented the model’s predictive performance. Additionally, the observational nature of our study limits the causal interpretation of our findings on pharmacotherapy. Finally, we lacked data on other potentially effective treatments, such as psychosocial interventions, which might influence mortality risk in FEP.

## Conclusions

This prognostic study developed and validated an ML model to predict mortality risk in FEP. Our findings suggest that if further validated, the model could help to develop personalized interventions to mitigate mortality risk. Prospective trials are required to confirm the clinical validity of the approach to stratify pharmacotherapy based on the predicted risk, and future studies should investigate the effectiveness of pharmacoptherapies not examined in the present study, such as clozapine, in different ML model–based risk groups.

## Notes

Supplement 1.
eMethods.
**eTable 1.** Variables Used for Training the Machine Learning Model to Predict Death Within 2 Years After First-Episode Psychosis (FEP) Diagnosis and Their Corresponding Importance (Gain) to the Model **eTable 2.** Machine Learning Classification Performances for the Prediction of Mortality Across the Study Samples **eTable 3.** Differences in the Patterns of Use of Different Oral Antipsychotics and Long-Acting Injectable Antipsychotics (LAIs; Grouped Together) Among Those Predicted to Die and to Survive in the Discovery Sample **eTable 4.** Association Between Use vs Nonuse of Medications and Risk of Death in Between-Individual Analysis in the Swedish Discovery Sample (n = 20 000) **eFigure 1.** Flowchart Depicting the Model Development and Validation Analyses in the Present Study **eFigure 2.** Proportion of Correct Out-of-Training Predictions Among Different Causes of Death in the Discovery Sample **eFigure 3.** Calibration Plots for the Model Predictions in the 2 Validation Samples **eFigure 4.** Association of Different Pharmacotherapies and the Risk of Death in the Discovery Sample (n = 20 000) Without Machine Learning–Based Stratification

Supplement 2.
Data Sharing Statement
