# Supplementary material for: Development and Validation of a Machine Learning–Based Model of Mortality Risk in First-Episode Psychosis
Source: JAMA Netw Open. 2024 Mar 18;7(3):e240640. doi: 10.1001/jamanetworkopen.2024.0640 (PMC10949098; doi:10.1001/jamanetworkopen.2024.0640)
Supplement: Supplement 1. — eMethods. eTable 1. Variables Used for Training the Machine Learning Model to Predict Death Within 2 Years After First-Episode Psychosis (FEP) Diagnosis and Their Corresponding Importance (Gain) to the Model eTable 2. Machine Learning Classification Performances for the Prediction of Mortality Across the Study Samples eTable 3. Differences in the Patterns of Use of Different Oral Antipsychotics and Long-Acting Injectable Antipsychotics (LAIs; Grouped Together) Among Those Predicted to Die and to Survive in the Discovery Sample eTable 4. Association Between Use vs Nonuse of Medications and Risk of Death in Between-Individual Analysis in the Swedish Discovery Sample (n = 20 000) eFigure 1. Flowchart Depicting the Model Development and Validation Analyses in the Present Study eFigure 2. Proportion of Correct Out-of-Training Predictions Among Different Causes of Death in the Discovery Sample eFigure 3. Calibration Plots for the Model Predictions in the 2 Validation Samples eFigure 4. Association of Different Pharmacotherapies and the Risk of Death in the Discovery Sample (n = 20 000) Without Machine Learning–Based Stratification [file jamanetwopen-e240640-s001.pdf]

Supplementary Online Content

Lieslehto J, Tiihonen J, Lähteenvuo M, et al. Development and validation of a machine learning–based model of mortality risk in first-episode psychosis. *JAMA Netw Open*. 2024;7(3):e240640. doi:10.1001/jamanetworkopen.2024.0640

eMethods.

eTable 1. Variables Used for Training the Machine Learning Model to Predict Death Within 2 Years After First-Episode Psychosis (FEP) Diagnosis and Their Corresponding Importance (Gain) to the Model

eTable 2. Machine Learning Classification Performances for the Prediction of Mortality Across the Study Samples

eTable 3. Differences in the Patterns of Use of Different Oral Antipsychotics and Long-Acting Injectable Antipsychotics (LAIs; Grouped Together) Among Those Predicted to Die and to Survive in the Discovery Sample

eTable 4. Association Between Use vs Nonuse of Medications and Risk of Death in Between-Individual Analysis in the Swedish Discovery Sample (n = 20 000)

eFigure 1. Flowchart Depicting the Model Development and Validation Analyses in the Present Study

eFigure 2. Proportion of Correct Out-of-Training Predictions Among Different Causes of Death in the Discovery Sample

eFigure 3. Calibration Plots for the Model Predictions in the 2 Validation Samples

eFigure 4. Association of Different Pharmacotherapies and the Risk of Death in the Discovery Sample (n = 20 000) Without Machine Learning–Based Stratification

This supplementary material has been provided by the authors to give readers additional information about their work.

eMethods.

1.1. Datasets

We identified the Swedish cohort using data from the National Patient Register (inpatient and specialized outpatient care) and the MiDAS Register (disability pensions and sickness absence). In the Swedish cohort, we utilized the following registers: the National Patient Register (all hospital care periods and specialized outpatient visits with diagnoses from July 2005 to December 2021), the Prescribed Drug Register (prescription drug purchases from July 2005 to December 2021), the Causes of Death Register (causes of death from 2006 to 2021), and the LISA register (demographic characteristics).

The Finnish cohort was identified from the Hospital Discharge Register maintained by the National Institute of Health and Welfare. Data for the Finnish cohort were collected from the Hospital Discharge Register (all hospital care periods with diagnoses from 1972 to 2017), the Prescription Register (reimbursed prescription drug purchases from 1995 to 2017), and the Causes of Death Register from Statistics Finland (1972–2017).

1.2. Machine Learning Pipeline

We used XGBoost (https://xgboost.ai/about) to train our model in the discovery sample using all available 51 variables. None of the variables used for training the model had over 20% missing values, and none of the participants had >40% missing values. We did not impute missing values due to XGBoost's ability to handle missing values. We trained the model within a nested cross-validation framework (eFigure 1). Nested cross-validation is a technique used to evaluate a machine learning model's out-of-training performance while also tuning the hyperparameters. In this method, there are two levels of cross-validation: the outer loop splits (10 folds, five permutations) the data into training and testing sets, and the inner loop selects the best hyperparameters (i.e., hyperparameter tuning, three folds). Nested cross-validation helps avoid overfitting and provides an accurate estimate of the model's generalizability. We tuned the following hyperparameters: eta/learning_rate (2^{-8}, 2^{-6}, 2^{-4}, 2^{-2}), gamma/min_split_loss (2^{-16}, 2^{-6}, 2^{2}), max_depth (3,5,7), the number of decision trees (100, 300, 500) and min_child_weight (2^{-16}, 2^{-6}, 2^{4}, 2^{8}). These hyperparameters were optimized using grid search and by employing maximal balanced accuracy (i.e., an average of sensitivity and specificity) as a selection criterion within the nested cross-validation framework. We set subsample and colsample_bytree parameters as 0.5. Also, we set scale_pos_weight as the ratio of actual deaths and survivals over the follow-up in the discovery sample to counteract class imbalance that could lead to imbalanced predictions. Other hyperparameters were left default. Model significance was assessed by comparing observed performance (i.e., AUROC) with predictions based on 5,000 random label permutations of the outcome label (i.e., mortality within two years).

We determined variable importance by calculating its feature gain, which measures the fractional contribution of each variable (feature) to the model. The higher the gain for a variable, the more critical it is for the model's prediction. Each variable's average gain in the discovery sample is provided in eTable 2.

We then aimed to identify a small set of the most relevant variables. For this purpose, we retrained another XGBoost model in the Swedish discovery sample using only the top 10% most important (i.e., five) variables. The hyperparameter tuning was again conducted within the cross-validation framework as described above. We then applied the model to independent Swedish and Finnish validation samples.

We visualized the variable contribution to this parsimonious model using SHAP (SHapley Additive exPlanations), a game-theoretic approach that facilitates the explanation of machine learning model predictions. SHAP works by calculating the "importance" of each feature, taking into account the magnitude of the effect and the interaction between features. It then uses game theory to allocate the contribution (positive or negative) of each feature to the final prediction.

© 2024 Lieslehto J et al. *JAMA Network Open.*

eTable 1. Variables Used for Training the Machine Learning Model to Predict Death Within 2 Years After First-Episode Psychosis (FEP) Diagnosis and Their Corresponding Importance (Gain) to the Model

**Variable Name**	**Importance (Gain)**
The number of different substance use disorder comorbidities (ICD-10: F10-19) year before the FEP diagnosis	0.131
Age at FEP (years)	0.121
The total number of previous somatic (i.e., non-psychiatric) hospitalizations (two years before the FEP diagnosis)	0.120
Duration of the first hospitalization due to psychosis in days. The variable is set to zero for those diagnosed at an outpatient clinic.	0.108
Male gender (yes/no)	0.073
Income from work during a previous calendar year before FEP (brutto, as Kronor)	0.057
The sum of unemployment days during the previous calendar year before FEP	0.043
Has a parent with schizophrenia (ICD-10: F20 or F25) (yes/no)	0.041
Whether the person had a specialized outpatient visit due to SUD within three months after FEP (yes/no)	0.037
Family situation: Single without children (yes/no)	0.034
Education: university/college (i.e., >12 years) education (yes/no)	0.028
Whether the person had a specialized outpatient visit due to psychosis within three months after FEP (yes/no)	0.027
Family situation: youth (<=20 years) living at home (with their parents) (yes/no)	0.023
The number of continuous days on sick leave a year before FEP	0.020
Born in Sweden (yes/no)	0.018
Diagnosis of FEP during inpatient visit (yes/no)	0.018
Use of antipsychotics during 30 days after cohort entry (yes/no)	0.017
Born outside of Europe (yes/no)	0.016
Residence: densely populated city (yes/no)	0.011
The severity of major depressive disorder one year before FEP	0.010
Education: only elementary level (i.e., nine years) education (yes/no)	0.010
Residence: rural areas (yes/no)	0.009
Education: high-school level (i.e., 10-12 years) education (yes/no)	0.008
FEP diagnosis of Unspecified Psychosis, ICD-10: F29 (yes/no)	0.007
FEP Diagnosis of Brief Psychotic Disorder, ICD-10: F23 (yes/no)	0.007
Whether the person has visited a psychiatric hospital a year before the FEP(yes/no)	0.007
Benzodiazepines during 30 days after cohort entry (N03AE01 clonazepam, N05BA, N05CD) (yes/no)	0.007
Residence: towns and suburbs (yes/no)	0.007
Substance-induced psychosis year before FEP (yes/no)	0.007
Antidepressants during 30 days after cohort entry (N06A) (yes/no)	0.006
Family situation: single with children (yes/no)	0.005
Family situation: married or cohabitant with children (yes/no)	0.005
FEP diagnosis of Delusional Disorder, ICD-10: F22 (yes/no)	0.004
Olanzapine oral N05AH02 during 30 days after cohort entry (yes/no)	0.004
Family situation: married or cohabitant without children (yes/no)	0.004
Suicide attempt (ICD-10: X60-X84, Y10-Y34) year before FEP (yes/no)	0.004
Aripiprazole oral N05AX12 during 30 days after FEP (yes/no)	0.004
Quetiapine oral N05AH04 during 30 days after cohort entry (yes/no)	0.004
Any LAI during 30 days after FEP (yes/no)	0.004
Mood stabilizer use during 30 days after FEP (yes/no)	0.004
Any employment during the previous calendar year before FEP (yes/no)	0.003
SUD drug use during 30 days after cohort entry (N07BB, N07BC) (yes/no)	0.003

© 2024 Lieslehto J et al. *JAMA Network Open.*

FEP diagnosis of Schizophrenia, ICD-10: F20 (yes/no)	0.003
Born in Europe (outside of Sweden) (yes/no)	0.003
Personality disorder (ICD-10: F60-F69) comorbidity year before FEP (yes/no)	0.002
ADHD drug use during 30 days after FEP (yes/no)	0.001
FEP diagnosis of schizoaffective disorder, ICD-10: F25 (yes/no)	0.001
FEP diagnosis of other psychotic disorder ICD-10: F28 (yes/no)	0.001
Granted disability pension within three months of FEP (yes/no)	0.000
FEP diagnosis of schizotypal personality, ICD-10: F21 (yes/no)	0.000
FEP diagnosis of shared psychotic disorder, ICD-10: F24 (yes/no)	0.000

© 2024 Lieslehto J et al. *JAMA Network Open.*

eTable 2. Machine Learning Classification Performances for the Prediction of Mortality Across the Study Samples

	TP	TN	FP	FN	AUROC	Sens %	Spec%	BAC%
**Two-Year Mortality Prediction:**								
Discovery Sample (N=20,000): trained on all features (out-of-training predictions)	209	14,001	5649	141	0.71	59.71	71.25	65.48
Discovery Sample (N=3992): restricted to those without any antipsychotic treatment during two years after baseline	58	2812	1095	27	0.74	68.24	71.97	70.10
Discovery Sample (N=16,008): restricted to those with some antipsychotic treatment during two years after baseline	151	11,189	4554	114	0.70	56.98	71.07	64.03
Validation sample 1 (Sweden, N=4052): Predictions using the final model (5 variables) trained in the discovery sample	37	3037	947	31	0.70	54.44	76.23	65.32
Validation sample 2 (Finland, N=1490): Predictions using the final model (5 variables) trained in the discovery sample	20	1051	408	11	0.67	64.52	72.04	68.28
**15-Year Mortality Prediction:**								
Discovery Sample (N=590): trained on all features (out-of-training predictions)	21	432	114	23	0.69	47.73	79.12	63.42
Validation sample 1 (N=113): Predictions using the final model (5 variables) trained in the discovery sample	4	88	9	12	0.67	30.77	88.00	59.38
Validation sample 2 (N=742): Predictions using the final model (5 variables) trained in the discovery sample	53	494	163	32	0.69	62.35	75.19	68.77
**20-Year Mortality Prediction:**								
Validation sample 2 (N=122): Predictions using the final model (5 variables) trained in the discovery sample	9	82	21	10	0.66	47.37	79.61	63.49

Abbreviations: TP=true positive, TN=true negative, FP=false positive, FN=false negative, AUROC=area under the receiver operating characteristic curve, Sens%=sensitivity%, Spec%=specificity%, BAC%=balanced accuracy.

eTable 3. Differences in the Patterns of Use of Different Oral Antipsychotics and Long-Acting Injectable Antipsychotics (LAIs; Grouped Together) Among Those Predicted to Die and to Survive in the Discovery Sample

Treatment	Predicted to Die		Predicted Survival		Statistical Testing
	Users	Percentage	Users	Percentage	
Any LAI	914	15.60	2235	15.80	χ^2 = 0.11, P-value = 0.738
Aripiprazole	1234	21.07	4201	29.71	χ^2 = 155.83, P-value < 0.0001
Olanzapine	2994	51.11	7212	51.00	χ^2 = 1.08, P-value = 0.298
Quetiapine	1391	23.75	3056	21.61	χ^2 = 10.81, P-value = 0.001
Risperidone	955	16.30	3348	23.67	χ^2 = 132.86, P-value < 0.0001
Polypharmacy	2492	42.54	6416	45.37	χ^2 = 13.30, P-value = 0.0003

Abbreviation: LAI=Long-Acting Injectable

© 2024 Lieslehto J et al. *JAMA Network Open.*

eTable 4. Association Between Use vs Nonuse of Medications and Risk of Death in Between-Individual Analysis in the Swedish Discovery Sample (n = 20 000)

Treatment	Medication Group	Predicted to Die		Predicted Survival	
		HR	95%CI	HR	95%CI
Any LAI	Antipsychotic	0.45	0.23-0.88	0.84	0.50-1.40
Aripiprazole	Antipsychotic, oral	0.77	0.47-1.26	0.38	0.20-0.69
Olanzapine	Antipsychotic, oral	0.97	0.72-1.31	0.77	0.53-1.11
Quetiapine	Antipsychotic, oral	1.16	0.77-1.73	0.95	0.59-1.53
Risperidone	Antipsychotic, oral	0.65	0.35-1.21	0.38	0.18-0.82
Polypharmacy	Antipsychotic, oral	0.83	0.59-1.18	1.02	0.73-1.44
Any Mood Stabilizer	Mood Stabilizer	0.64	0.46-0.90	1.03	0.75-1.40
Any Antidepressant	Antidepressant	1.22	1.00-1.49	1.06	0.84-1.34
Any Benzodiazepine	Benzodiazepine	1.79	1.45-2.21	2.16	1.66-2.81

Abbreviations: HR=Hazard Ratio, 95% CI=95% Confidence Interval and LAI=Long-Acting Injectable

© 2024 Lieslehto J et al. *JAMA Network Open.*

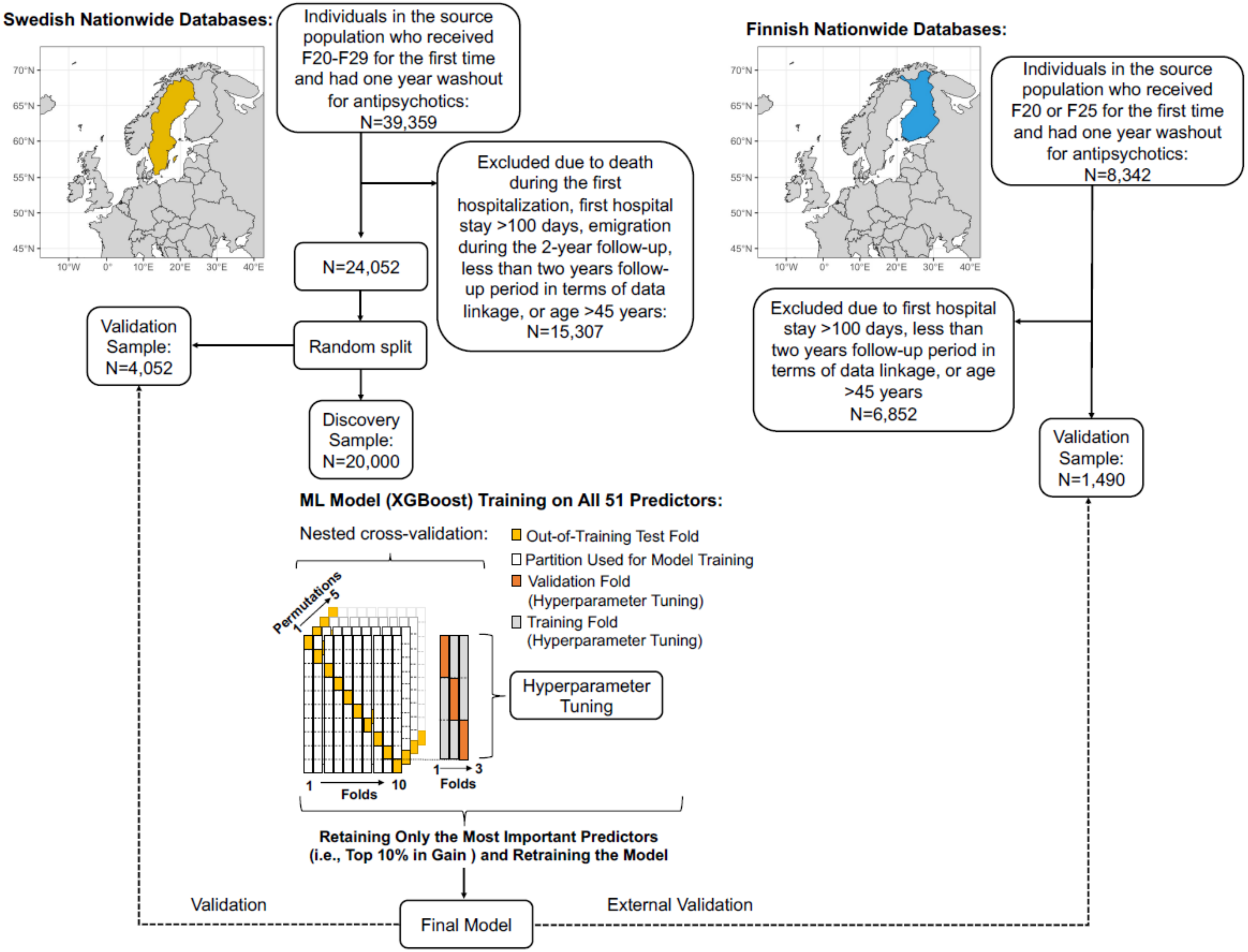

eFigure 1. Flowchart Depicting the Model Development and Validation Analyses in the Present Study

© 2024 Lieslehto J et al. *JAMA Network Open.*

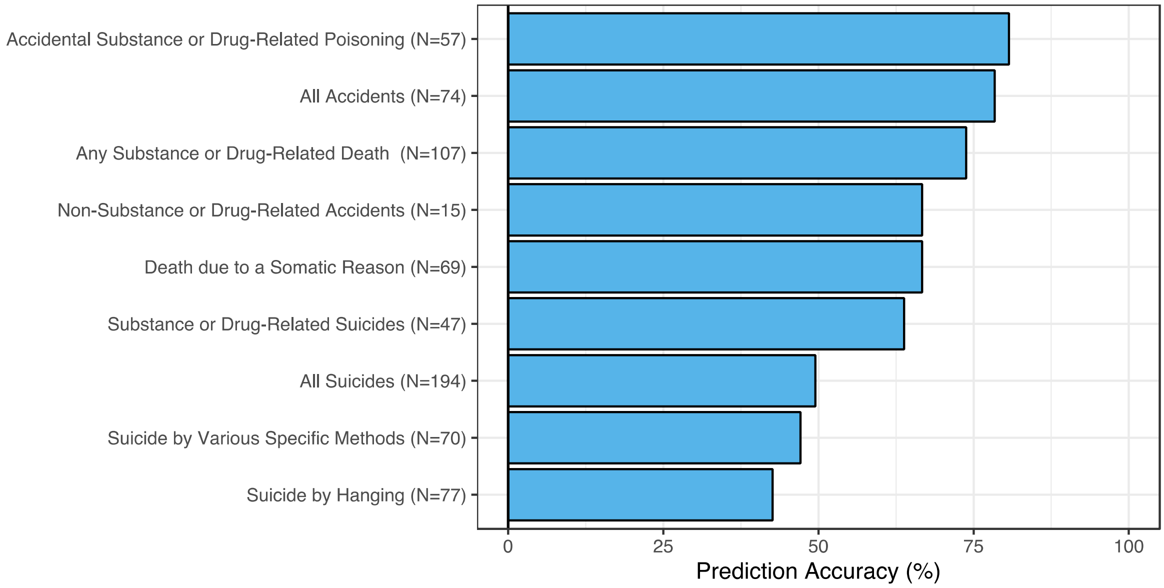

eFigure 2. Proportion of Correct Out-of-Training Predictions Among Different Causes of Death in the Discovery Sample

Accidental Substance or Drug-Related Poisonings (ICD-10 codes: X40-X45), All Accidents (V01-V99, W00-W99, X00-X59), Any Substance or Drug-Related Death (F10-F19, X40-X45, X60-X65, Y10-Y15), Non-Substance or Drug-Related Accidents (V01-V99, W00-99, X46-X59), Death due to a Somatic Reason (A00-R99, except for F00-F99 diagnoses), Substance or Drug-Related Suicides (X60-X65, Y10-Y15), All Suicides (X60-X84, Y10-Y34), Suicide by Various Specific Methods (X66-X69, X71-X84, Y10-Y19, Y21-Y34) and Suicide by Hanging (X70 and Y20).

© 2024 Lieslehto J et al. *JAMA Network Open.*

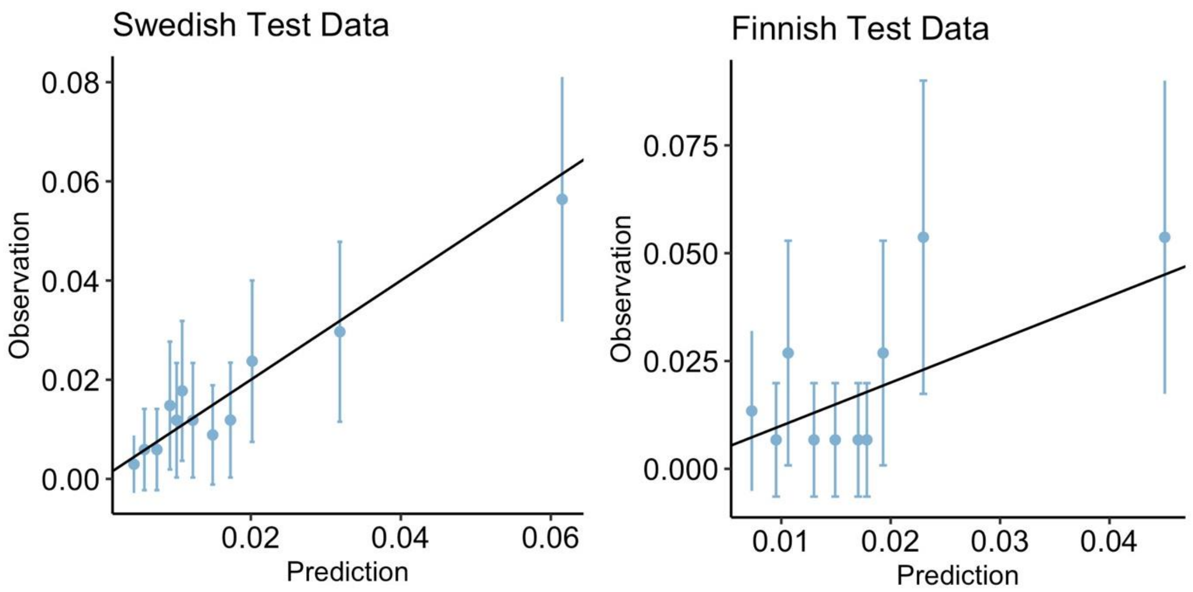

eFigure 3. Calibration Plots for the Model Predictions in the 2 Validation Samples

Calibrations were conducted using Platt scaling (logistic regression). The calibration model was trained in the discovery sample and applied to Swedish and Finnish validation samples. Calibration performance for the Swedish validation sample (Brier score=0.016, intercept=-0.149, slope=0.966) and the Finnish validation sample (Brier score=0.020, intercept=0.048, slope=0.970).

© 2024 Lieslehto J et al. *JAMA Network Open.*

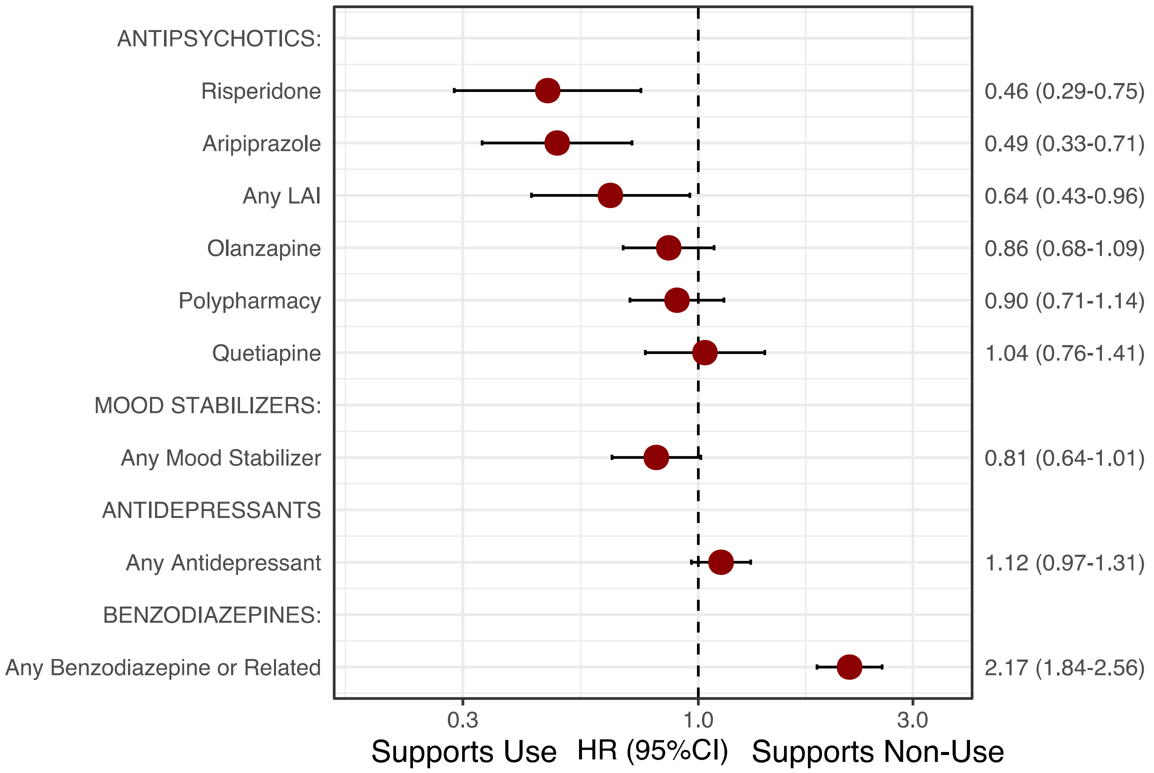

eFigure 4. Association of Different Pharmacotherapies and the Risk of Death in the Discovery Sample (n = 20 000) Without Machine Learning–Based Stratification

Antipsychotics are oral except for "any LAI," which includes all long-acting injectable antipsychotics.

© 2024 Lieslehto J et al. *JAMA Network Open.*
