# Supplementary material for: Development and Validation of a Machine Learning–Based Model of Mortality Risk in First-Episode Psychosis
Source: JAMA Netw Open. 2024 Mar 18;7(3):e240640. doi: 10.1001/jamanetworkopen.2024.0640 (PMC10949098; doi:10.1001/jamanetworkopen.2024.0640)
Supplement: Supplement 2. — Data Sharing Statement [file jamanetwopen-e240640-s002.pdf]

Data Sharing Statement

Lieslehto. Development and Validation of a Machine Learning–Based Model of Mortality Risk in First-Episode Psychosis. *JAMA Netw Open*. Published March 18, 2024.
doi:10.1001/jamanetworkopen.2024.0640

Data
Data available: No

Additional Information
Explanation for why data not available: The data used in this study cannot be made publicly available due to privacy regulations. According to the General Data Protection Regulation, the Swedish law SFS 2018:218, the Swedish Data Protection Act, the Swedish Ethical Review Act, and the Public Access to Information and Secrecy Act, these types of sensitive data can only be made available for specific purposes, including research, that meet the criteria for access to this sort of sensitive and confidential data as determined by a legal review. Readers may contact Professor Kristina Alexanderson (kristina.alexanderson@ki.se) regarding the data.
